# Histone Acetylation and Modifiers in Renal Fibrosis

**DOI:** 10.3389/fphar.2022.760308

**Published:** 2022-04-26

**Authors:** Fengchen Shen, Shougang Zhuang

**Affiliations:** ^1^ Department of Nephrology, Shanghai East Hospital, Tongji University School of Medicine, Shanghai, China; ^2^ Department of Medicine, Rhode Island Hospital and Alpert Medical School, Brown University, Providence, RI, United States

**Keywords:** histone acetylation, histone acetyltransferases, histone deacetylases, bromodomain and extraterminal proteins, renal fibrosis, epithelial-mesenchymal transition, fibroblast activation, inflammatory response

## Abstract

Histones are the most abundant proteins bound to DNA in eukaryotic cells and frequently subjected to post-modifications such as acetylation, methylation, phosphorylation and ubiquitination. Many studies have shown that histone modifications, especially histone acetylation, play an important role in the development and progression of renal fibrosis. Histone acetylation is regulated by three families of proteins, including histone acetyltransferases (HATs), histone deacetylases (HDACs) and bromodomain and extraterminal (BET) proteins. These acetylation modifiers are involved in a variety of pathophysiological processes leading to the development of renal fibrosis, including partial epithelial-mesenchymal transition, renal fibroblast activation, inflammatory response, and the expression of pro-fibrosis factors. In this review, we summarize the role and regulatory mechanisms of HATs, HDACs and BET proteins in renal fibrosis and provide evidence for targeting these modifiers to treat various chronic fibrotic kidney diseases in animal models.

## Introduction

Chronic kidney disease (CKD) has become a worldwide health problem, with increased incidence rate and poor prognosis. According to statistics, the global prevalence of CKD is 13.4%, and 10.6% of CKD patients are in stage 3–5 (Hill et al., 2016). Progression of CKD to end-stage renal disease (ESRD) is a costly and important clinical event with substantial morbidity (Hill et al., 2016), which is driven by persistent fibrotic response. Therefore, understanding the mechanism by which renal fibrosis is developed is essential for delaying or halting the progression of CKD to ESRD.

Development and progression of renal fibrosis grows out of a number of pathological processes, including partial epithelial-mesenchymal transition (pEMT), activation of pericyte/resident fibroblasts, becoming myofibroblasts, inflammation, extracellular matrix (ECM) deposition (Liu, 2011). Many studies have confirmed that pEMT involves epithelial cell arrest at the G2/M phase of cell cycle, resulting in production of growth factors and cytokines that promote renal pericyte/fibroblast activation and overproduction of ECM components in the interstitium (Wynn, 2008). Proinflammatory cell infiltration into the injured kidney further promotes the deposition of ECM, damaging renal structure and function (Meran and Steadman, 2011). Under pathological conditions, myofibroblasts also release large amounts of matrix proteins, leading to an imbalance in matrix protein secretion and degradation (Eyden, 2005; Kramann et al., 2013; Humphreys, 2018). Furthermore, M1-type macrophages infiltrated to the interstitum are able to be transformed to a M2-type that acquires an ability to promote renal fibrosis by generating some profibrotic factors such as transforming growth factor -β1 (TGF-β1) (Anders and Ryu, 2011; Tang et al., 2019). Interestingly, inhibition of HDAC activity is shown to reduce infiltration of M1 and M2a macrophages and promote their conversion to M2c macrophages, and ultimately alleviate renal fibrosis (Tseng et al., 2020). Although the factors and mechanism responsible for the development of renal fibrosis remain incompletely clear, expression of genes and activation of signaling pathways associated with renal fibrosis has been proved to be regulated by multiple posttranslational modifications, including histone acetylation (Morgado-Pascual et al., 2019; Rousselle et al., 2021).

Acetylation is a process that transfers an acetyl functional group from one molecule (i.e., acetyl coenzyme A) to another. It can occur in both histone and non-histone proteins (Narita et al., 2019; Xia et al., 2020). Coenzyme A is a key intermediate metabolite whose specificity severely affects the activity of histone acetylation. In other words, CoA, as a substrate for acetylation, influences the level of downstream Histone H3 acetylation in the nucleus and cytoplasm (Sivanand et al., 2018). Histones are a group of proteins abundant in lysine and arginine residues in the nucleus, and act as spools around which DNA winds to create nucleosome. Histone acetylation can weaken the binding between histones and DNA, making DNA more accessible to the transcription machinery, thereby promoting gene transcription. Through a similar process, some non-histone proteins (ie., NF-kB, STAT3) are also acetylated in mammalian cells (Narita et al., 2019; Xia et al., 2020). Acetylation of non-histone proteins affects protein function by regulating several molecular mechanisms such as protein stability and enzymatic activity, as well as controlling protein–protein and protein–DNA interactions (Narita et al., 2019; Xia et al., 2020).

Protein acetylation is a dynamic process that is controlled by histone acetyltransferases and histone deacetylases. Histone acetyltransferases (HATs) catalyze the transfer of an acetyl group from a donor molecule to a target lysine residue, leading to protein acetylation, while histone deacetylases (HDACs) catalyze removal of acetyl groups from lysine residues, causing protein deacetylation (Verdin and Ott, 2015; Wu et al., 2020). Moreover, acetylation is regulated by a family of the bromodomain and extra-terminal (BET) proteins, which can recognize the acetylated lysine in histones and other proteins (Cochran et al., 2019). As the “readers” of lysine acetylation, bromodomain proteins interact with the site-specifically acetylated nucleosomes and are responsible for transducing the signal carried by acetylated lysine residues (de la Cruz et al., 2005). Therefore, the occurrence of protein acetylation and exertion of its functional role need a fine coordination of HATs, HDACs, and BET proteins: HATs and HDACs are responsible for determining the dynamic changes of acetylation of the targeted proteins (Filgueiras et al., 2017) while BET proteins are required for translating the acetylated proteins to exert biological functions (Cochran et al., 2019). In the past four decades, acetylation and its modifiers, including HATs, HDACs and BET proteins, have been extensively investigated and found to be involved in the pathogenesis of various diseases such as cancer, cardiovascular disease, and neurological diseases. Recent studies have also demonstrated the role of these acetylation modifiers in the development and progression of renal fibrosis (Li et al., 2016; Morgado-Pascual et al., 2019; Nie et al., 2020).

In this review, we summarize the role and mechanism of histone acetylation and its modifiers, HAT, HDAC and BET proteins, in the regulation of renal fibrogenesis.

### HATs and Renal Fibrosis

HATs are divided into three categories, including general control non-derepressible5 (Gcn5)-related N-acetyltransferases (GNATs) superfamily, p300/CBP, and MYST proteins (Lee and Workman, 2007). GNAT superfamily is composed of four sequence motifs, which can specifically bind to substrates in cells. The members of GNATs superfamily include Gcn5, PCAF, Elp3, Hpa2, and Hat1. All of them are involved in chromatin remodeling and gene transcription (Sterner and Berger, 2000). p300/CBP proteins not only have intrinsic HAT activity, but are also transcription co-activators that work with other transcription factors such as STAT proteins, AP1, and NF-kB to activate gene transcription (Shiama, 1997). MYST proteins include Esa1, Sas2, as3mTip60, MOF, MOZ, MORF, and HBO1. All the HATs have been shown to regulate cell cycle, transcriptional silencing and chromatin acetylation, but their exact mechanisms are still not fully understood (Sterner and Berger, 2000). Selective HAT inhibitors have been used to investigate the role of some HATs in renal fibrosis and the mechanisms involved (Table 1).

**TABLE 1:** Effects of HAT inhibitors on renal fibrosis.

HAT inhibitor	Target	Model	Effects and mechanisms	References
L002	p300	Ang II-induced hypertension, renal tubular epithelial cells	Inhibit renal fibrosis; reduced deposition of ECM components; inhibit EKR1/2 and Smad2 signaling pathways	Rai et al. (2017), Yang et al. (2015)
C646	p300	Renal tubular epithelial cells	Prevent the development of EMT; reduce TGF-β_1_-induced phosphorylation of Smad3	Yang et al. (2015)
C66	p300	Diabetic nephropathy	Inhibit renal fibrosis; Inhibit JNK activation; suppress CTGF, FN and PAI-1 gene transcription	Wang et al. (2015)
garcinol	PACF	UUO	Inhibit renal fibrosis; inhibit the activation of NF-κB and Nrf2; decrease the expression of HO-1, NQO-1, catalase and SOD1	Chung et al. (2016), Chung et al. (2019)

HAT, histone acetyltransferase; ECM, extracellular mextrix; EKR1/2, mitogen-activated protein kinase 1/2; EMT, Epithelial–mesenchymal transition; TGF-β_1_, Transforming growth factor; JNK, c-Jun N-terminal kinase; CTGF, connective tissue growth factor; F, fibronectin; PAI-1, plasminogen activator inhibitor 1; PACF, P300/CBP-associated factor; UUO, unilateral ureteral obstruction; NF-κB, Nuclear factor kappa B; Nrf2, Nuclear factor-erythroid factor 2-related factor 2; HO-1, Heme oxygenase-1; NQO-1, NADPH, Quinone acceptor Oxidoreductase 1; superoxide dismutase type 1.

Extensive studies have demonstrated that p300/CBP is closely related to the development and progression of renal fibrosis. P300/CBP is highly expressed in the kidney in a murine model of angiotensin II-induced renal fibrosis and in cultured rat renal tubular epithelial cells (Rai et al., 2017; Rai et al., 2019). C646 is a highly selective p300 inhibitor that reduces collagen IV and α-SMA expression in renal tubular epithelial cells *in vitro* (Ni et al., 2014). Its anti-fibrotic effect has also been recently demonstrated in an animal model of diabetic nephropathy, showing that C646 could effectively inhibit the release of pro-fibrotic mediators and deposition of ECM proteins in diabetic kidneys. The antifibrotic effect of C646 was associated with reducing oxidative stress and blocking pro-inflammatory responses and pro-fibrotic pathways (Lazar et al., 2021). Administration of L002, another p300 inhibitor, also blunted histone acetylation and reduced deposition of ECM components in the hypertensive kidneys (Rai et al., 2017). Interestingly, the anti-fibrotic effect of L002 was not related to blood pressure control, but associated with the inhibition of EKR1/2 and Smad2 signaling pathways (Rai et al., 2017). In cultured renal tubular epithelial cells, overexpression of p300 also increased STAT3 acetylation on Lys685 while inhibition of p300 by siRNA C646 reduced TGF-β_1_-induced phosphorylation of Smad3 and prevented the development of EMT (Yang et al., 2015). Moreover, in animal models of diabetic kidney disease, treatment with C66, a curcumin analogue that has an ability to inhibit p300 activity, also protected the kidney by inhibiting JNK activation and suppressing CTGF, FN-1, and PAI-1 gene transcription (Wang et al., 2015). Importantly, renal protection continued to be observed 3 months after C66 treatment (Wang et al., 2015). In addition to its anti-fibrotic effect in the kidney, L002 and C646 have been shown to inhibit cardiac hypertrophy (Sunagawa et al., 2011; Xiao et al., 2016; Rai et al., 2019), pulmonary fibrosis, liver fibrosis and systemic sclerosis (Ghosh et al., 2013; Yao et al., 2018; Sanders et al., 2020). These findings suggest that HATs play an important role in mediating tissue fibrosis.

P300/CBP-associated factor (PCAF) also has histone acetyl transferase activity and regulates the molecular machinery leading to renal fibrosis and inflammation. Chung et al., demonstrated that PCAF is highly expressed in mouse kidneys after unilateral ureter obstruction (UUO) and is coincident with activation of NF-κB signaling and nuclear decline of anti-inflammatory factor Nrf2 Chung et al. (2019). Both P300 and PACF can catalyze the acetylation of lysine-310 of RelA to activate the NF-κB signaling pathway (Ikeda et al., 2000). Thus, inactivation of p300 and PACF enzyme activities may provide a way to inhibit NF-κB (Sheppard et al., 1999). In this context, it has been reported that NF-κB and Nrf2 interact with each other to regulate renal inflammation and renal injury; the PCAF inhibitor garcinol is effective in altering these cellular responses by downregulating histone acetylation levels (Chung et al., 2019). It should be noted that Nrf2 is not only an anti-inflammatory factor, but also has antioxidative properties. Antioxidant enzymes such as HO-1, NQO-1, catalase and SOD1 are downstream signaling molecules of Nrf2 and are involved in renal oxidative stress (Chung et al., 2016). In the UUO model, Nrf2 was activated, and the expression levels of HO-1, NQO-1, catalase and SOD1 were increased; garcinol treatment can inhibit these responses (Chung et al., 2016; Chung et al., 2019). Thus, Nrf2 is well recognized as a responsive center of PCAF that drives inflammation and oxidative stress in the kidney. Additionally, PCAF may drive the EMT and renal fibrosis by changing the nuclear localization of NF-kB (Chung et al., 2019).

In contrast to p300/CBP and PCAF, Tip60, a member of the MYST family of acetyltransferases, has been reported to protect against tissue fibrosis. This is evidenced by the observation that depletion of Tip60 increases the level of interstitial fibrosis in the heart of mice (Fisher et al., 2016). Currently, the role of Tip60 and other members of the MYST family of acetyltransferases in renal fibrosis has not been reported and needs further investigations.

### HDACs and Renal Fibrosis

HDACs are a family composed of a total of 18 members that are divided into four categories. Class I HDACs includes HDAC1, 2, 3, and 8; Class II HDACs are divided into two subclasses: Class IIa (HDAC4, 5, 7, and 9) and class IIb (HDAC6, 10). Class III HDACs are a large family, including SIRT1-7. Class IV HDAC has only one member–HDAC11 (Ma et al., 2016). While Sirtuins require nicotinamide adenine dinucleotide (NAD+) for their catalytic activity, the class I, II, and IV family HDACs are Zn^2+^-dependent enzymes that differ in their cellular localization, expression and catalytic domains. The role of SIRT1-7 in kidney disease has been recently reviewed in detail (Morigi et al., 2018; Hong et al., 2020); the current review focuses only on the role and mechanisms of class I, II, IV classes of HDACs in the pathogenesis of renal fibrosis (Table 2).

**TABLE 2:** Effect of HDAC inhibitors or gene deletion on renal fibrosis.

HDAC inhibitors or knockout mice	Target	Model	Effects and mechanisms	References
TSA	Class I/II HDACs	UUO	Inhibit EMT; down regulate the TGF-β_1_ expression; inhibit the JNK/Notch-2 signaling pathways	Ma et al. (2009), Tung et al. (2017)
MS-275	Class I HDACs	UUO	Suppress phosphorylation of Smad3, EGFR and STAT3; reduce the expression of TGF-β_1_	Liu et al. (2013)
RGFP966; HDAC3 knock-out mice	HDAC3	UUO, AAN	Depress the klotho expression	Chen et al. (2021)
PCI34051	HDAC8	UUO	Inhibit the development of EMT; preserve expression of BMP-7 and Klotho	Zhang et al. (2020)
MC1568, HDAC4 siRNA	HDAC4	UUO	Reduce the expression of pro-fibrosis factors, TGF-β_1_/Smad3 and NF-κB; preserve expression of klotho, BMP-7 and Smad7	Xiong et al. (2019)
Tubastatin A, HDAC6 siRNA	HDAC6	Ang II-induced hypertension	Inhibit the transcription of TGF-β, Smad3, collagen I, and CTGF.	Choi et al. (2015)
ACY-1215	HDAC6	UUO	Inhibit the activation of TGF-β_1_/Smad3, EGFR/AKT, STAT3 and NF-κB signaling pathways	Chen et al. (2020)
Quisinostat; HDAC11 siRNA	HDAC11	UUO, Ang II-induced hypertension	Suppress expression of Kruppel-like factor 15	Mao et al. (2020)

HDAC, histone deacetylase; TSA, Trichostatin A; UUO, unilateral ureteral obstruction; EMT, Epithelial–mesenchymal transition; TGF-β1, Transforming growth factor; JNK, c-Jun N-terminal kinase; EGFR, epidermal growth factor receptor; STAT3, Signal transducer and activator of transcription 3; BMP-7, Bone morphogenetic protein 7; NF-κB, Nuclear factor kappa B.

Growing evidence indicates that HDACs are involved in the regulation of renal fibrosis. Initial studies demonstrated that administration of trichostatin A (TSA), a pan inhibitor of class I and II HDACs, significantly inhibited EMT and attenuated renal fibrosis by downregulation of TGF-β_1_ expression and inhibition of the JNK/Notch-2 signaling pathways (Ma et al., 2009; Tung et al., 2017), as well as preservation of E-cadherin expression (Yoshikawa et al., 2007). MS-275, a selective inhibitor of class I HDACs (Srivastava et al., 2010) was shown to inhibit EMT of renal epithelial cells by suppressing phosphorylation of Smad3, EGFR, and STAT3 (Liu et al., 2013). Furthermore, treatment with MS-275 reduced the expression of TGF-β_1_ in UUO injured kidneys (Liu et al., 2013). These data suggest that both class I and class II HDACs play a role in mediating renal fibrosis.

Isoform-selective inhibitors of HDACs have been developed over the past decade, allowing investigation into the functional role of individual HDACs in renal fibrosis. Recent studies have identified three isoforms of class I HDACs- HDAC2, HDAC3, and HDAC8 as therapeutic targets of renal injury. HDAC2 has been shown to induce apoptosis of renal tubular epithelial cells through suppression of BMP-7 in acute kidney injury (Ma et al., 2017) and mediate renal fibrosis in diabetic mice (Noh et al., 2009; Zheng et al., 2022). HDAC3 was elevated in fibrotic kidneys following UUO and aristolochic acid nephropathy (AAN). Pharmacological inhibition of HDAC3 with RGFP966, a selective inhibitor of HDAC3, or genetic deletion of HDAC3, attenuated the renal fibrosis (Chen et al., 2021). The antifibrotic effect of HDAC3 inhibition is associated with restoration of Klotho, a renoprotective protein, in the kidney, suggesting that HDAC3 can drive renal fibrogenesis through depression of Klotho (Chen et al., 2021). Moreover, HDAC8 was upregulated in the kidney following UUO injury in a time-dependent manner, which was coincident with increased expression of three fibrotic markers: α-smooth muscle actin, collagen 1 and fibronectin and deposition of collagen fibers in the injured kidney (Zhang et al., 2020). Treatment with PCI34051, a highly selective inhibitor of HDAC8, largely reduced the expression of those fibrotic responses and restored expression of acetyl-cortactin, a target of HDAC8, during injury following UUO injury (Zhang et al., 2020). Mechanistic studies revealed that blocking HDAC8 inhibited activation of multiple signaling pathways associated with the development of EMT (Smad3, STAT3, β-catenin) and preserved expression of both BMP-7 and Klotho (Zhang et al., 2020). Since HDAC8 is primarily expressed in renal epithelial cells, but not renal interstitial fibroblasts, HDAC8 may induce renal fibrosis by triggering EMT and release of profibrotic growth factors/cytokine, subsequently leading to renal fibroblast/pericyte activation and renal fibrosis. This is suggested by the observations that siRNA-mediated silencing of HDAC8 also suppresses expression of profibrotic markers: α-SMA, collagen I and fibronectin in cultured renal epithelial cells (Zhang et al., 2020).

As indicated above, class II HDACs are subdivided into class IIa and IIb. In an early study, HADC4 has been shown to be involved in fibrosis secondary to diabetic nephropathy (Wang et al., 2014). Since HADC4 is one of class IIa HDAC isoforms, we have recently examined the role and mechanisms of class IIa HDACs and HDAC6 in the development of renal fibrosis following UUO injury. We found that class IIa HDACs (4, 5, 7, 9) are expressed in renal epithelial cells following UUO (Xiong et al., 2019). Administration of MC1568, a class IIa HDAC inhibitor, reduced renal fibrosis and inhibited serum and TGF-β_1_-induced of EMT in cultured renal epithelial cells (Xiong et al., 2019). Mechanically, MC1568 treatment inhibited phosphorylation of Smad3, NF-κB, and up-regulation of integrin ɑVβ6 and largely preserved expression of klotho, BMP-7 and Smad7, three proteins associated with renal protection, in kidneys injured by UUO (Xiong et al., 2019). In cultured renal epithelial cells, MC1568 treatment and siRNA-mediated HDAC4 silencing also inhibited expression of several profibrotic markers, including α-SMA, fibronectin and collagen 1, as well as phosphorylation of Smad3 and NF-κB (Xiong et al., 2019). Another study showed that HDAC4 silencing attenuated glomerular fibrosis and inhibited expression of the aformentioned fibrotic proteins in diabetic mice (Raval et al., 2021). Thus, we suggest that among class IIa HDACs, HDAC4 may play a predominant role in promoting renal fibrosis. Due to lack of an inhibitor for individual class IIa HDACs, it is impossible to clarify the role of each isoform of this family in regulating renal fibrosis and molecular mechanisms involved in animal models. Further studies are needed to detail the role of class IIa HDAC isoforms *in vivo* using genetic approaches.

HDAC6 is a most studied isoform of class IIb HDACs in renal diseases. Unlike other HDAC isoforms, whose deletion in mice leads either to death *in utero* or severe developmental defects, mice with HDAC6 deletion develops normally without major organ dysfunction (Zhang et al., 2008). This unique feature of HDAC6 may have important implications for the safety of potential therapeutic inhibition of HDAC6. In the past decade, several highly selective HDAC6 inhibitors have been developed, Tubastatin A (TA), has been found to be effective in improving polycystic kidney disease (ADPKD) (Cebotaru et al., 2016), hypertensive nephropathy (Choi et al., 2015), acute kidney injury (AKI) (Shi et al., 2017) and peritoneal fibrosis (Xu et al., 2017) in animal models. In angiotensin II-induced hypertension models, down-regulation of HDAC6 by TA or siRNA could inhibit the transcription of fibrosis related genes, such as TGF-β, Smad3, collagen I, and CTGF (Choi et al., 2015). Our recent studies have shown that blockade of HDAC6 with ACY-1215 also attenuated renal fibrosis through a mechanism involving the inactivation of TGFβ1/Smad3, EGFR/AKT, STAT3 and NF-κB signaling pathways in a murine model of UUO injury (Chen et al., 2020). These data suggest that HDAC6 could be a potential therapeutic target for the treatment of renal fibrosis. Although HDAC10, another isoform of class IIb HDACs, has also been reported to be elevated in the kidney after UUO injury (Choi et al., 2016), its role in renal fibrosis remains unclear due to lack of HDAC10-specific inhibitor(s).

HDAC 11 is the sole isoform of class IV HDACs. A recent study revealed that HDAC11 was also expressed in the kidney of murine models of CKD induced by UUO, Ang II or a high-fat diet. Administration of quisinostat, a non-specific HDAC11 inhibitor, attenuated UUO-induced renal fibrosis and reduced Ang II-induced profibrotic response in cultured renal epithelial cells. Similar inhibition on Ang II-induced profibrotic response was also observed in cultured renal epithelial cells with depletion of HDAC11 by siRNA. Mechanistical studies demonstrate that HDAC11 contributes to renal fibrosis by suppressing expression of Kruppel-like factor 15, an anti-fibrogenic factor (Mao et al., 2020).

In summary, it seems that all three classes of Zn2+-dependent HDACs are involved in the development of renal fibrosis, based on experimental results obtained using class -selective inhibitors. Only HDAC3-depleted mice were used to confirm its anti-fibrotic effects. Due to the possible off-target effects of various HDAC inhibitors, further studies require to use mice with deletion individual HDAC to verify the role of HDAC isoforms in renal fibrosis.

### BET Protein and Renal Fibrosis

BET proteins are composed of Brd2, Brd3, Brd4, and Brdt and act as an epigenetic reader (de la Cruz et al., 2005; Morgado-Pascual et al., 2019). Brd2, Brd3, and Brd4 are ubiquitously expressed but Brdt is only expressed in the male germ cell. Structurally. BET proteins contain two brominated domains, an extra-terminal domain (ET) and a C-terminal domain (CTD) (Morgado-Pascual et al., 2019). The ET domain is highly conserved and responsible for recruiting proteins to activate transcription (Rahman et al., 2011), and CTD domains is responsible for recruiting the positive transcription elongation factors (P-TEFB) to the transcriptional complex. Besides histones, BET proteins can also interact with acetylated lysine residues in other proteins such as transcription factors to regulate their functions (Cochran et al., 2019). Emerging evidence indicates that BET proteins can regulate many cellular functions, including cell growth, differentiation, inflammation and pericyte/fibroblast activation (Stathis and Bertoni, 2018; Morgado-Pascual et al., 2019) (Table 3).

**TABLE 3:** Effects of BET inhibitors on renal fibrosis.

BET inhibitor	Target	Model	Effects and mechanisms	References
JQ1; Brd4 siRNA	BET, Brd4	UUO	Prevent the inflammatory response; dephosphorylation of NF-κB and/or other transcriptional factors/co-factors	Suarez-Alvarez et al. (2017), Zhou et al. (2017)
JQ1	BET	Ang II-induced hypertension	Inhibit EMT; decrease the expression of collagen III, α-SMA and vimentin	Wang et al. (2019)
I-BET151	BET	UUO	Inhibit the activation of fibrogenic cytokines, profibrotic signaling pathways, transcription factors and growth factor receptors; reduce cell arrest in G2/M phase	Xiong et al. (2016)
Brd4 siRNA	Brd4			
I-BET151	BET	Hyperuricemic nephropathy	Reduce expression of TGF-β1; inhibit the dephosphorylation of Smad3, ERK1/2 and NF-kB; suppress inflammatory response	Xiong et al. (2021)

BET, bromodomain and extraterminal; UUO, unilateral ureteral obstruction; Brd4, Bromodomain containing 4; NF-κB, Nuclear factor kappa B; EMT, Epithelial–mesenchymal transition; α-SMA, α-Smooth muscle actin; TGF-β1, Transforming growth factor; ERK1/2, mitogen-activated protein kinase ½.

The most widely investigated BET proteins is Brd4. In addition to recruiting the p-TEFB complex to the promoter region of genes to activate transcription, Brd4 can bind to NF-κBp60 at acetylated lysine-310 residue to sustain nuclear NF-κB activation, leading to increased gene expression of some inflammatory factors such as CCL-2 and IL-17A. Treatment with JQ1, a specific inhibitor that blocks the interaction between Brd4 bromine domain and lysine residue (Shahbazi et al., 2016), prevents the inflammatory response (Suarez-Alvarez et al., 2017). Given that the NF-κBp60-medaited inflammatory response is pivotal in renal damage and fibrosis following various insults, the anti-inflammatory effects of BET inhibitors may lead to attenuation of renal fibrosis. Indeed, it has been observed that in the experimental model of UUO-induced renal damage, or immune mediated glomerulonephritis, BET inhibition by JQ1 markedly reduced renal fibrosis, which is coincident with dephosphorylation of NF-κBp60 and/or other transcriptional factors/co-factor such as STAT3 and Smad3 (Zhou et al., 2017). In a mouse model of hypertension-induced renal fibrosis, JQ1 can also inhibit the development of EMT, as indicated by decreased expression of collagen III, α-SMA and vimentin (Wang et al., 2019). In addition, JQ1 treatment significantly reduced serum creatinine and urea nitrogen levels, as well as expression of renal injury markers NGNAL and Kim-1 in the model of renal injury induced by cisplatin and angiotensin (Suarez-Alvarez et al., 2017; Sun et al., 2018). Mechanistically, JQ1-elicidated inhibition of BET proteins can increase the expression of antioxidant genes Nrf2 and HO-1, thus maintaining the redox balance of the kidney (Sun et al., 2018).

Another BET protein inhibitor, I-BET151, has also been observed to be effective in alleviating renal damage and fibrosis in a murine model of UUO (Xiong et al., 2016). The anti-fibrotic effect of I-BET151 was associated with inactivation of several profibrotic signaling pathways, including STAT3, NF-kB, and Smad3 (Xiong et al., 2016). Moreover, I-BET151 was effective in inhibiting the activation of transcription factors and growth factor receptors, such as c-Myc, P53, EGFR, and PDGFR, and reduced cell cycle arrested at G2/M phase (Xiong et al., 2016). Recently, we further examined the effect of I-BET151 on the development of hyperuricemic nephropathy (HN) in a rat model and found that expression of Brd2 and Brd4, but not that of Brd3 was elevated in the injured kidney (Xiong et al., 2021). Treatment with I-BET151 significantly prevented renal dysfunction, decreased urine microalbumin, and attenuated renal fibrosis. Furthermore, I-BET151 reduced expression of TGF-β1, inhibited dephosphorylation of Smad3 and ERK1/2 and NF-kB, and suppressed inflammatory response in the kidney (Xiong et al., 2021). Although we detected an increase in serum levels of uric acid and xanthine oxidase, an enzyme that catalyzes production of uric acid, and a decrease in the expression of renal organic anion transporter 1 and 3, which promote urate excretion in the model of HN, I-BET151 treatment did not affect these responses (Xiong et al., 2021). These data suggest that Brd proteins may be involved in the mechanism leading to renal inflammation and fibrosis, but not associated with alteration of serum uric acid levels.

Interestingly, the BET protein inhibitor abapetalone has entered clinical trials and proved to be effective in improving the prognosis of CKD (Wasiak et al., 2018). By measuring plasma samples from patients with CKD stage 4 or 5 after treatment, researchers demonstrated that abapetalone significantly decreased plasma IL-6, NF-κB and proatherosclerotic factor, suggesting that abapetalone can reduce the incidence of CKD complications (Wasiak et al., 2018). These clinical data on abapetalone suggest the feasibility of BET protein inhibitors in improving the prognosis of CKD.

In conclusion, BET protein could induce renal fibrosis through multiple mechanisms, such as promoting the expression of proinflammatory and pro-fibrotic cytokine/growth factors and activation of some signaling molecules and transcriptional factors. Although the precise mechanism of BET protein-mediated renal fibrosis remains incompletely understood, studies from animal models of CKD and a clinical trial in patients with CKD have provided evidence for therapeutic potential of BET inhibitors in the treatment of renal fibrosis and associated cardiovascular events in CKD.

## Conclusion and Perspectives

It is widely accepted that histone acetylation participates in the pathophysiological mechanism of renal fibrosis. During the process of histone acetylation, HATs and HDACs regulate histone acetylation by transferring coenzyme A acetyl to or from lysine residues, while BET proteins are responsible for recognizing acetylated lysine residues. Histone acetylation has been shown to be involved in the regulation of various pathological processes in fibrotic kidney diseases, including pEMT, pericyte/fibroblast activation, ECM deposition, inflammation, and oxidative stress. Preclinical studies have proven beneficial effects of some inhibitors for HAT, HDAC, and BET proteins in acute and chronic renal damage. A clinical trial has also demonstrated the beneficial effect of the small molecule BET inhibitor apabetalone in improving kidney function and the prognosis in patients with diabetes mellitus type 2 and with cardiovascular diseases of high risk. These data suggest that targeting acetylation may be a promising approach for the treatment of CKD.

At present, class I, II, and III HDACs are widely recognized to be involved in various kidney diseases (Table 4), and some of them have been verified in human tissues. Specially, four HDAC inhibitors, Vorinostat, Romidepsin, Panobinostat, and Belinostat, have been approved by the United States Food and Drug Administration (FDA) for certain hematological cancers, clinical trials are needed to assess their efficacy in the treatment of human chronic fibrotic kidney diseases. Although inhibition of HATs and HDACs results in an opposite effect on acetylation, preclinical studies have proved evidence that both HAT and HADC inhibitors exert anti-fibrotic effects. A possible explanation is that HATs such as p300 have other functions (i.e., activation of NF-κB) in addition to their acetyltransferase activity. Given that NF-kB or some other transcriptional factors are critical regulator of inflammation in renal fibrosis, inhibition of p300 may not only interrupt its acetyltransferase activity, but also inflammatory responses, thereby inhibiting renal fibrosis. Another explanation is that currently used HAT inhibitors lack specificity and resultant anti-fibrotic effects may be due to their inhibition on other profibrotic mechanisms. Thus, it has become important to further study and understand the fundamental functions of individual HATs in renal fibrosis by using genetic approaches (i.e., knockout mice) and develop more specific HAT inhibitors.

**TABLE 4:** Expression of HDACs in human kidney diseases.

Human kidney disease	HDAC inhibitors	Target	References
Focal segmental glomerulosclerosis	Valproic acid	class I HDAC	Van Beneden et al. (2013)
	TSA	class I/II HDAC	Van Beneden et al. (2013)
	Vorinostat	class I HDAC	Inoue et al. (2019)
	RGFP966	HDAC3	Liu et al. (2016)
IgA nephropathy	Valproic acid	class I HDAC	Dai et al. (2016)
	TSA	class I/II HDAC	Dai et al. (2016)
Diabetic nephropathy	Valproic acid	class I HDAC	Khan et al. (2015)
	TSA	class I/II HDAC	Noh et al. (2009)
	SK-7041	class I HDAC	Noh et al. (2009)
	Sodium butyrate	class I/II HDAC	Wu et al. (2018)
Polycystic kidneys	TSA	class I/II HDAC	Livingston et al. (2019)
	ACY-1215	HDAC6	Yanda et al. (2017)

HDAC, histone deacetylase; TSA, Trichostatin A.
